# Research gaps identified in Iran’s health technology assessment reports

**DOI:** 10.1186/s12961-023-01043-0

**Published:** 2023-12-11

**Authors:** Farideh Mohtasham, Bahareh Yazdizadeh, Mohammadreza Mobinizadeh

**Affiliations:** 1 Knowledge Utilization Research Centre (KURC), Tehran University of Medical Sciences, Kargar St., Keshavarz Blvd, P.O. Box 1417993357, Tehran, Iran; 2 National Institute for Health Research, Tehran University of Medical Sciences, Tehran, Iran

**Keywords:** Technology assessment, Biomedical, Research, Iran

## Abstract

**Introduction:**

Identifying gaps in the evidence is a useful byproduct of conducting a health technology assessment (HTA). This study aims to identify research gaps in Iran’s HTA reports.

**Method:**

We reviewed the HTA reports published between 2014 and 2016. Then, we developed two separate questionnaires for principal investigators (PIs) and independent HTA researchers. The questionnaire for independent HTA researchers consisted of four main parts. However, the PIs’ questionnaire consisted of two main parts. We also conducted a literature search in the PubMed database in November 2017 to find frameworks for prioritizing research gaps. We also conducted a semi-structured interview with the head of the Iran’s HTA Office at that time and sought feedback based on his expert opinion about questionnaires, the priority-setting tool and our process for extracting research gaps.

**Results:**

A total of 11 HTA reports published between 2014 and 2016 by Iran’s HTA Office were selected for the study. Of these 11 reports, 5 involved technologies related to medical equipment, while 6 involved medical and surgical interventions. Assessing the outcomes of technology use in various indications and updating HTAs when new evidence arises; evaluating the viewpoints of patients, clinicians and key technology users; conducting post-marketing evaluations of technology; comparing the impact of the technology in question to other treatments for the same condition; and requesting long-term clinical and cost–effectiveness data for technologies with limited follow-up periods were identified as the main gaps by independent HTA researchers and PIs.

**Conclusions:**

The research gaps identified from Iran’s HTAs could be utilized by research funding agencies.

## Introduction

To make better-informed decisions about improving health, an important question is: what evidence is needed to support these decisions? [1].

Calls for the use of evidence-based decision-making (EBDM) in the policy-making process have increased, aiming to achieve state and national objectives for improved population health with a greater focus on it. EBDM is a process that utilizes the best available scientific evidence regarding the effectiveness of various programs or policies and translates that evidence into real-world practice by incorporating community-level data, resources and priorities [2].

Evidence-informed policy-making is also about using the best available research evidence to help make policy decisions [3]. In recent years, several interventions have been implemented to enhance EBDM in Iran. These include the solicitation of applied research proposals, the allocation of 2% of medical universities’ funds to health service research, the promotion of capacity-building for the production and adoption of systematic reviews and practice guidelines, and the establishment of a Health Technology Assessment (HTA) Unit within the Ministry of Health and Medical Education (MOHME) [4]. The HTA is a tool to assist evidence-based decision-making (EBDM) and support evidence-informed health policy-making. The definition of it is:

"Health Technology Assessment (HTA) is a multidisciplinary process that employs explicit methods to evaluate the value of a health technology at various stages of its lifecycle." The purpose is to inform decision-making in order to promote an equitable, efficient, and high-quality health system [5].

HTAs in Iran have been established since 2007 and was first introduced as a secretariat by the Deputy of Health at the Ministry of Health and Medical Education (MOHME). Since 2010, the HTA unit has collaborated with the National Institute of Health Research (NIHR)[6].

NIHR is an organization dedicated to health research and the development of evidence-informed policy-making. The organization produces national request for proposals (RFPs) for HTA projects in response to assignments from the MOHME.

NIHR utilizes HTA guides such as the Standard Framework of Full Health Technology Assessment (fHTA), Rapid Technology Assessment (RTA) and Technology Snapshot (TSS) for Diagnostic Technologies and Therapeutic Technologies. These guides include a set of questions[7] based on The HTA Core Model®[8].

The government sponsors and funds HTA activities in Iran.

Scientific Committee of HTA Department within the Ministry of Health is responsible for setting priorities for HTA requests and undertaking assessments. It utilizes an explicit process for priority-setting. Burden of disease, frequency of the clinical condition, healthcare cost, political concern and public and media concern are some important criteria that our HTA organization uses to set priorities. By conducting external reviews of the reports, their quality is ensured.

Electronic and printed versions of reports, websites and papers published in national scientific journals are the most commonly used methods or activities to disseminate reports.

The target users of the reports include policy-makers, consumer associations, healthcare professionals, patients and representatives of the clinical disciplines impacted by each health technology.

Reports such as assessment reports are types of products that HTA organizations produce.

Assessment reports are documents that result from the assessment process. They are based on systematic reviews and revisions of scientific evidence, with a focus on aspects such as the efficacy, safety, effectiveness and efficiency of medical technologies.The assessment is adapted to the specific health scenario being analysed and is dependent on the requirements of the commissioning organization [9].

So, an HTA must always be firm in its research and scientific approach. So, having access to relevant research is a cornerstone of HTAs.

Some of the stakeholder questions in HTA reports remain unanswered due to a lack of evidence or poor quality [10].

The difference between the research-generated information that is needed in the policy-making process and what is currently available can be called the research gap [11].

So, in our study, if the questions from NIHR’s frameworks for HTA reports are not answered or if the answers are of low quality, or if questions outside of the list of established frameworks are created in the mind of the reader of the reports, HTA research gaps will be identified.

This study aims to identify research gaps in Iran’s HTAs and develop a process for extracting research questions from them.

## Methods

We reviewed the HTA reports published between 2014 and 2016.

The proposals and final reports were received from the NIHR.

Then, we developed two separate questionnaires for principal investigators (PIs) and independent HTA researchers. These questionnaires were based on the pilot project that aimed to distil researchable questions from the research gaps identified in the HTA reports of the Alberta Heritage Foundation for Medical Research (AHFMR) [12].

So, independent HTA researchers assessed the adherence of HTA reports to the questions of the defined frameworks, as well as the quality of the responses obtained. They also raised questions that lingered in their minds after reading the reports.

The PIs also provided reasons for not finding answers to certain research questions. They also identified questions that remained in their minds about that technology after the assessment.

To pilot the questionnaires, three HTA reports were randomly selected and completed by two individuals, and their final opinion on the necessary edits for each questionnaire was applied in consensus with a third person.

Finally, the questionnaire for independent HTA researchers consisted of four main parts.

1. Is, methodologically, the quality of the HTA reports based on the instructions provided by the NIHR? Did the proposal and the final report adhere to the defined structure?
2. What are the critical questions that each selected HTA report must answer? How well are these questions answered, and what are the reasons for not answering them? What were the questions that needed to be answered in the research proposal? What questions are answered in the final report? What are the answers to the questions in the final report? How do you evaluate the quality of the obtained response? If the questions remain unanswered, what is the reason for not answering them?
3. In the text of the final report, what questions were raised entitled “There is a need for further studies”?
4. What questions did remain in the mind of the independent HTA researcher after reading the report?

In the section on adherence to the defined structure, it was asked whether the proposal and final report included searches in Cochrane databases, PubMed, Embase and the Trip Database. It was also asked whether a separate search strategy was determined for each database, whether the quality of included studies was determined using relevant checklists such as the Critical Appraisal Skills Program (CASP) [13] and the International Network of Agencies for Health Technology Assessment (INAHTA) [14], whether a flow diagram was determined according to the preferred reporting items for systematic reviews and meta-analysis (PRISMA) [15], and whether the final report complied with the principles of preparation and reporting of HTA projects, specifically the HTA core model [8].

The PIs’ questionnaire also consisted of two main parts.

1. What are the critical questions that each selected HTA report must answer? How is the quality of answering these questions assessed, and what are the reasons for not answering them? What were the questions you were supposed to answer at the beginning of the research? What steps did you take to find the answers to each of the questions? Did you find the answers to the questions? How do you evaluate the quality of the obtained response? If the questions remain unanswered, what is the reason for not answering them?
2. What questions remained in the minds of the project team at the end of the report?

Two independent HTA researchers (FM and MM), who are responsible for conducting the HTA reports and were not involved in any aspect of the selected HTA report’s production, reviewed the reports, completed the questionnaire and identified the research gaps for each document.

We also identified PIs who wrote selected HTAs. If it was unclear who the PI was, or if we were unable to contact them, we reached out to the other researchers who were involved in producing the report. Each PI was contacted via email and telephone.

Independent HTA researchers and PIs were asked to complete questionnaires for each report within 1 month. After the deadline, reminders were sent to them.

We compare tabulated research gaps identified from the perspectives of two independent HTA researchers and PIs and descriptively generate lists of research gaps for each HTA report. We also conducted a literature search in the PubMed database in November 2017 to find frameworks for prioritizing research gaps. Our search strategy was as follows:

("prioritise" OR "priority setting" OR "priority-setting" OR "setting of priorit*") AND ("Health policy") AND ("research")

The time period considered was from 2010 to 2017, and the language was restricted to English.

After reviewing the titles of the selected articles, we chose the ones that were relevant to our research topic for further study. We then proceeded to examine the full text of these selected articles.

Once we developed our questionnaires, identified research gaps and found a priority-setting tool, we conducted a semi-structured interview with the head of the MOHME’s HTA Office at that time and sought feedback based on his expert opinion. We asked him to review our process and provide feedback on its clarity and potential ease of use. We also asked him to provide general comments and suggestions for specific items that might need to be added, removed or reworded.

A face-to-face interview was conducted with the head of MOHME’s HTA Office at his workplace. At the beginning of the interview, an explanation was given about the objective of the study, and verbal consent was obtained to record the interview. The interviewee was assured that the audio recording would be stopped whenever he deemed necessary. He was then asked to review our process for identifying research gaps and our designated tool for prioritizing them and to provide feedback on their clarity and potential ease of use.

At the end of the interview, the interviewer asked additional questions and discussed points that had not been mentioned or had only been briefly mentioned, taking into account the tool’s questions. Tehran University of Medical Sciences’ research ethics committees evaluate the ethical soundness of our submitted proposal and approve it.

## Results

### Specifications of HTA reports

Between 2014 and 2016, the National Institute of Health Research of Iran (NIHR) published 11 HTA reports in response to assignments from the MOHME.

The most frequently assessed health technology (HT) was medical devices.

The topic areas covered included medical-related technologies and alternative treatments for mapping brain activity, treating skin wrinkles and acne scars, diagnosing breast cancer, measuring bone density, treating heart failure, relieving chronic back pain, methods of skin rejuvenation and treating major depressive disorder.

The kind of organizations that were asking for reports has not been specified. Most of the HTA reports produced national recommendations. The main target audience to whom they addressed the recommendations were policy-makers.

Several of the HTA reports identified future research gaps/needs in their discussion section. These gaps include areas where there are no or limited precise studies, particularly in the field of economic studies. Other areas of concern include low-quality studies or inconsistencies between the studies that were included. Additionally, there is insufficient information on important subgroups, outcomes or comparative interventions. Lastly, there is a lack of long-term follow-up studies in certain areas.

Two PIs had two projects and seven PIs had one project. So then, we sent the questionnaire to nine PIs. Of the questionnaires sent to the PIs, seven researchers replied, resulting in a response rate of 77.78%. Therefore, we were able to identify research questions from the perspective of reporting researchers for eight projects.

### Identifying research gaps from independent HTA researcher’s perspective

Two independent HTA researchers read the reports, answered the questionnaire and extracted a list of research gaps for each report. These gaps were then reviewed and agreed upon in a third-person session. Research gaps, as identified from the perspective of independent HTA researchers, categorized into six main categories:

1. Some HTA reports have missed important indications. In addition to the specified indications in an HTA report, it is important to investigate other relevant indications or combinations. The lack of important indications in the reports should be investigated. In the assessment of two health technologies for breast cancer diagnosis, it was discovered that one of the technologies is also applicable to gastric cancer. Therefore, as a research gap, we can address the issue of whether that technology is suitable for other patient populations. Three of our HTAs could assess the outcomes of technology use in different populations.
2. Some HTA reports missed relevant studies due to limitations in language or time constraints. Restriction during the literature search stage may result in the exclusion of eligible studies, particularly those related to the origin of technology in languages other than English. In the evaluation of two health technologies in relieving chronic back pain, only English-language studies were included, limiting its search. However, it seems necessary to study papers in the Chinese language as well in that report because one of the technologies was an ancient Chinese method. Three of our HTAs could be reassessed without language and time restrictions at the stage of literature search.
3. Some HTA reports have methodology problems in conducting their analyses. For example, in evaluating the cost–effectiveness of two neuroimaging techniques for mapping brain activity, a discount rate of 3% was used. Maybe it is difficult to determine which discount rate to choose. So, a variety of discount rates and different assumptions should be used to reach the conclusions. Five of our HTAs could be repeated using appropriate and relevant methods.
4. Some HTA reports reach uncertain conclusions due to insufficient evidence and poor quality of evidence. About eight of our HTAs could benefit from updates when new evidence becomes available.
5. All of our reports address inquiries regarding the safety, clinical effectiveness and/or cost–effectiveness of health technologies. However, they did not take into account the legal, ethical, organizational, patients and social aspects. So, based on the importance of these research gaps to decision-makers, it is important to consider the need for assessing these aspects.
6. Some HTA reports assessed health technologies during the market approval process in Iran. The results are based on international evidence. Thus, there is often uncertainty about the transferability of the results and whether we can expect the same effect as in the studies if the technology is implemented in Iran. So, new HTAs could be applied post-marketing to determine the value of these technologies.

### Identifying research gaps from PIs’ perspective

PIs read the reports, answer the questionnaires and extracted a list of research gaps for their report.

Assessing the outcomes of technology use in various indications and updating HTAs when new evidence becomes available were mentioned by PIs. Additionally, the PIs mentioned the importance of assessing the views of patients, clinicians and major consumers of technology, as well as evaluating technology at the post-marketing stage. They are also concerned with the brief length of follow-up periods in many studies that may not uncover potential effects. Therefore, they are requesting long-term clinical and cost–effectiveness data for these technologies. They highlighted a lack of research on the impact of the questioned technology in comparison to other treatments for the same condition as well. Main gaps from the perspective of independent HTA researchers and PIs are presented in Table 1.

**Table 1:** Main gaps from the perspective of independent health technology assessment (HTA) researchers and principal investigators (PIs)

Title of HTAs	Main gaps
Comparison of two health technologies in breast cancer	Reviews have found that this technology is also suitable for treating gastric cancer. Therefore, as a research gap, we can address the issue of whether this technology is suitable for patient populations - It was limited to English-language studies. It may be necessary to reassess it without any language restrictions - The discount rate of 7% was used. It could be repeated with different assumptions and discount rates
Comparison of two neuroimaging techniques for mapping brain activity	- The report reached uncertain conclusions due to a lack of evidence. Updates could be undertaken when new evidence becomes available - The report mentioned other relevant indications. It is important to investigate those indications - At the time of the project, the device was not available in Iran, so the costs had to be estimated. A new HTA could be conducted during the post-marketing phase of the technology
Evaluation of two health technologies in the treatment of skin wrinkles and acne scars	Due to the lack of appropriate evidence, great heterogeneity in studies and lack of appropriate evidence in the field of outcomes related to quality of life, the project failed to answer some questions such as: - What is the efficiency, effectiveness and cost–effectiveness of the technology compared with alternative technologies? - What is the experience and acceptance of patients and physicians regarding technology? - What is the lifespan of medical technology in patients?
Comparison of two health technologies in breast cancer diagnosis	- At the time of the project, the device was not available in Iran, so the costs were estimated. A new HTA could be applied during the post-marketing phase of technology - A threshold of three times the gross domestic product (GDP) per capita was used for the non-quality outcome. This was done to clarify the cost–effectiveness of the technology for policy-makers. Over time and with the completion of evidence, appropriate economic studies could be conducted
Evaluation of two health technologies in measuring bone density	- The report reached uncertain conclusions due to a lack of evidence and the low quality of the available evidence. Updates could be undertaken when new evidence is made available - At the time of the project, the device was not available in Iran, so the costs had to be estimated. A new HTA could be conducted during the post-marketing phase of the technology
Evaluation of two health technologies in treatment of patient with heart failure	- The report reaches uncertain conclusions due to a lack of evidence and the low quality of available evidence. Updates could be undertaken when new evidence becomes available - The report mentioned that the experience and acceptance of patients and physicians regarding technology are important. So, this question could be evaluated. - According to the report, the combined method is considered a more effective strategy compared with using each technology alone. Is there a need to conduct a separate study in this field?
Evaluation of two health technologies in relieving chronic back pain	- Only the cost–effectiveness results have been shown in the final report. So, the main question is: what is the safety and effectiveness of the technology? - Since one of the technologies originates from China, it seems necessary to also examine studies in the Chinese language. But the study limited its search to English-language studies. It seems necessary to reassess it without any language restriction - It is not clear who performed this method. What kind of professionals and what kind of patients can benefit from it? - The proposal mentioned that the economic analysis should be conducted from the society’s perspective. However, in the final report, it was conducted from the perspective the payers. The reason for this contradiction is not clear, and it is necessary to determine which perspective is more appropriate - At the time of the project, there were no head-to-head studies that examined the effectiveness and cost–effectiveness of these two treatment methods. Does the study need to be repeated to gather additional evidence of higher quality and accuracy?
Safety and efficacy assessment of a health technology in cancer treatment	- The aim of this project was to assess the safety and effectiveness. Is it necessary to conduct a study to assess the cost–effectiveness? - The limited number of studies involved the technology with other standard methods. Included studies are outdated and lack sufficient data to conduct appropriate quantitative analysis. Is it necessary to reassess it as soon as the relevant data becomes available?
Evaluation of two health technologies in the treatment of major depressive disorder	It is one of the project’s questions, but in the final report the cost-utility question was not addressed. Is it necessary to reassess it as soon as relevant data becomes available? - In the report, we have the following text: “There are statistics available on the prevalence of major depression in Iran. However, there is a lack of statistics specifically related to the prevalence of treatment-resistant depression. As a result, the statistics on the prevalence of major depression were used in the economic evaluation analysis.” - Is it necessary to design studies to identify the prevalence of treatment-resistant depression and then incorporate it into an economic evaluation analysis? - Regarding the clinical effectiveness of this method, the studies have yielded inconsistent results. Some studies confirm the effectiveness, while others do not. Does the study need to be repeated with greater sensitivity and precision to uncover additional evidence?
Evaluation of two health technologies in imaging systems	- Although the assessment of ethical, structural and social issues was requested, these questions remained unanswered without any explanation. Does the study need to be repeated to find the answers to these questions? - At the time of the project, the device was not available in Iran, so the costs had to be estimated. New HTAs could be conducted during the post-marketing phase of a technology
Safety, effectiveness and cost–effectiveness of one of the methods of skin rejuvenation	- The report reached uncertain conclusions due to a lack of evidence and the low quality of the available evidence. Updates could be undertaken when new evidence becomes available - The report mentioned some relevant findings. It is important to investigate those indications - The authors of the report believe that the use of microdermabrasion will become more widespread in Iran in the next few years compared with other technologies, thanks to its superior effectiveness and lower costs. New HTAs could be conducted to examine the use of technology and its outcomes

### Identifying the relevant tool to prioritize research gaps

Upon searching, we found 597 articles. Following the secondary screening and examination of the full texts of the articles, only one article remained that was relevant to prioritizing research gaps extracted from HTAs. That introduced the SPARK Tool to prioritize questions for systematic reviews in health policy and systems research [16].

Our inclusion criteria were to find a tool that prioritizes questions in health policy and systems research (HPSR) to address them in HTAs. In the item generation phase of the SPARK tool, an extensive literature search yielded 40 relevant articles. These articles were reviewed by the research team to create a preliminary list of candidate items for inclusion in the tool [16].

We also included tools that utilize inputs from both the supply side (HTA team) and the demand side (stakeholders).

The SPARK tool, which consists of 22 items, is divided into two modules. The first module consists of 13 items to be rated by policy-makers and stakeholders, while the second module consists of 9 items to be rated by systematic review teams [16].

Our recommended approach for administering the tool is for the HTA reviewer team to complete the module, which includes nine items. The team should rank these items based on their feasibility and appropriateness for conducting primary or review studies. The other module is then designed for policy-makers and stakeholders to prioritize questions based on their relevance.

### Expert opinion

About questionnaires, the priority-setting tool and our process for extracting research gaps, the head of the MOHME’s HTA Office, who is one of the founders of HTAs in Iran, stated the following in a 50-min face-to-face interview:

- "By evaluating other indicators of health technology, in addition to safety, effectiveness and cost–effectiveness, and by assessing questioned technology alongside other relevant routine interventions or combination thereof, we find a significant number of studies that many countries lack the capacity to conduct. Therefore, it is better to prioritize between them, that is, to question the research gaps that we anticipate encountering in the near future.
- Regarding the study design without language restrictions, based on my experience, I believe that we do not lose much by not implementing it because HTA studies are not widely conducted in countries where English is not the primary language.
- Finally, when conducting a study as a research centre, it is crucial to consider its sources … I might transition from one HTA study to another, but … I have to prioritize first, then confer to obtain the necessary funds".

He also believed that extracting research questions from HTA reports was an improvement. However, its use in Iran, a country grappling with the issue of HTAs, is questionable because it has no upstream rules.

## Discussion

This study aimed to identify research questions from HTA reports, compare the identified research questions from different perspectives and prioritize them.

Identifying the process of extracting and prioritizing research questions from HTA reports for the National Institute of Health Research and other organizations is the objective of this study.

Therefore, we developed two separate questionnaires: one for contacting principal PIs involved with evidence synthesis, and another for contacting two HTA experts. We asked them to identify research questions from HTA reports.

Based on the 11 HTA reports published by Iran between 2014 and 2016, we have identified a need for studies according to PIs and independent HTA reviewers:

- That have longer follow-up periods and without any restrictions on time or language.
- That assesses other indications of health technology.
- That updates existing HTAs when high-quality sufficient evidence becomes available and technology is introduced in Iran.
- That compares questioned technology with other relevant routine interventions.
- That conducts well-planned HTAs in situations where there is scientific uncertainty.
- That assesses additional aspects of health technologies other than common aspects.

After identifying the research gaps, our recommended process for prioritizing and formulating researchable questions is as follows: first, answer questions related to the appropriateness and feasibility of conducting a systematic review using SPARK Tool. Next policy-makers and stakeholders should answer questions regarding the relevance of the question to them.

The HTA reviewer team should complete the first module of the SPARK Tool to develop evidence maps of systematic reviews and of primary studies that address the relevant health technology. It would help to avoid questions that result in empty and duplicate reviews.

From our expert perspective, the SPARK tool has addressed technical capacity, but the crucial issue of financial capacity has not adequately considered. It would be beneficial to also inquire about how the project should be financed. Proposing the establishment of an HTA unit within research funding organizations, which would generate researchable questions based on the research gaps identified in HTA reports, could create a more advantageous scenario for connecting the identification of research gaps to the research funding process.

Our findings show that HTAs could be used as an untapped source of information to address research gaps. Existing research also declares that HTAs can identify gaps in evidence where there is a lack of quantity and/or quality research and methodological limitations in the existing research [17].

By identifying knowledge gaps, HTAs can encourage more research that will eventually improve healthcare [18]. In the impact assessment of Iran’s HTA study, after reviewing 23 HTA reports, it was found that several questions remained unanswered in a large number of reports. These questions include determining the disadvantages of using technology for professionals, society and the environment; determining the direct impact of technology on the quality of life, returning to work and daily activities; determining the impact of technology use on human dignity and its relationship with the cultural and religious beliefs of patients; determining the harmful effects of technology with different brands; and identifying ways to ensure the quality of technology use. Also, most of the reports have evaluated the technologies in terms of costs, and few have addressed other areas of evaluation [10]. In addition to confirming the existence of research gaps in Iran’s HTA reports, these findings emphasize the need to improve the HTA process in Iran.

The Swedish Agency for Health Technology Assessment and Assessment of Social Services (SBU) has a database of knowledge gaps in health technologies. These gaps pertain to technologies whose effects have been assessed with uncertainty or have not been sufficiently assessed in HTA reports due to insufficient evidence. The Swedish Research Council invites and grants applications that focus on addressing gaps identified in the SBU database [18].

The Division for Health Services of the Norwegian Institute of Public Health also publishes an annual report on research gaps identified through systematic reviews and HTAs. In 2015, the centre conducted 30 systematic review and HTA reports. Out of these, 24 reports indicated the need for more extensive and improved research. Specifically, they highlighted the need for research in the areas of other comparative arms of interventions, other indications and studies with long follow-up periods[19].

In Alberta, Canada, the Alberta Heritage Foundation for Medical Research (AHFMR) has also developed a promising pilot process for formulating researchable questions. It could work on a case-by-case basis to inform the research funding programs of the AHFMR about the research gaps identified by AHFMR HTA reports from the three stakeholder perspectives: health services research, clinical and policy. For example, the research gaps identified from an HTA report were the need for a standardized definition of the assessed technology, the need for research to determine which kind of patients do best with the assessed technology and the need to monitor outcome data and measure quality of life after using the assessed technology [12].

Belgium and the United Kingdom systematically review research gaps identified in HTA reports and offer research recommendations as part of their work. Their research recommendations are published, and high-priority topics are actively sent to research funding agencies [12]. In discussing the strengths and limitations of our research, it is important to note that the identification of research gaps in HTAs requires input from various stakeholders, including researchers, policy-makers, clinicians, consumers and the general public. Each of these groups may have different opinions regarding the need for future research [12]. However, our study was limited to only two perspectives. Although we planned to gather the viewpoints of stakeholders and policy-makers, unfortunately, despite our efforts, we were unable to access them.

In this study, our aim was to identify research gaps from HTAs and to develop a process for extracting them. But the data collection and search were limited to 6 years ago (in 2017). As a result, considering the time limit and the possibility of improving the quality of HTA reports in Iran over this period, the results must be interpreted with caution. It is important to focus more on the process of extracting research questions from HTAs, as well as other types of products and services provided by HTA organizations. We suggest rerunning it with HTA reports published up to 2023 for future research endeavours.

An important strength of our process is the identification of research gaps from the perspectives of HTA auditors and PIs of reports perspectives, and the prioritizing of these gaps using the SPARK tool.

It is unrealistic to distil researchable questions from the research gaps identified in all HTA reports. It is important to establish criteria for prioritizing HTA reports before attempting to extract research gaps.

In stating implications for practice and for research, we add that extracting research gaps from HTAs could serve as a Horizon Scanning [20] method for forecasting future trends and informing the development of technologies. It is an issue that could be considered in future research.

Due to a lack of high-quality clinical research evidence in most of our HTAs, there is a need to update them. Then, decisions about the timing of HTA updates are another important issue. When to update HTAs could be identified in future research by developing feasible and efficient approaches or tools.

## Conclusions

Our process facilitates the use of a systematic method for identifying research gaps in HTA reports. But a dedicated group needs to be identified to oversee the process from beginning to end.

We were unable to obtain stakeholders’ views in identifying research gaps. We therefore suggest identifying research gaps by engaging potential and relevant stakeholders in each HTA report. After aggregating research gaps from HTA auditors, principal investigators of the reports and stakeholders’ perspectives, we recommend prioritizing them using the SPARK Tool.

## Data Availability

The datasets used and/or analysed during the current study are available from the corresponding author on reasonable request.
